# Determinants of the optimal selection of vascular access devices: A systematic review underpinned by the COM‐B behavioural model

**DOI:** 10.1111/jan.16202

**Published:** 2024-05-02

**Authors:** Ismael Fernández‐Fernández, Enrique Castro‐Sánchez, Ian Blanco‐Mavillard

**Affiliations:** ^1^ Implementation, Research, and Innovation Unit Hospital de Manacor Manacor Spain; ^2^ Brunel University London Uxbridge UK; ^3^ National Institute for Health Research Health Protection Research Unit in Healthcare Associated Infection and Antimicrobial Resistance at Imperial College London London UK; ^4^ Global Health Research Group University of the Balearic Islands Palma Spain; ^5^ Hospital Regional Universitario de Malaga Malaga Spain; ^6^ Department of Nursing and Physiotherapy Universitat de les Illes Balears Palma Spain; ^7^ Care, Chronicity and Evidence in Health Research Group (CurES) Health Research Institute of the Balearic Islands (IdISBa) Palma Spain

**Keywords:** choice behaviour, nurse specialists, psychological models, specialization, vascular access devices, vascular access specialist team, vascular access team

## Abstract

**Background:**

Optimal selection of vascular access devices is based on multiple factors and is the first strategy to reduce vascular access device‐related complications. This process is dependent on behavioural and human factors. The COM‐B (Capability, Opportunity, Motivation, Behaviour) model was used as a theoretical framework to organize the findings of this systematic review.

**Methods/Aims:**

To synthesize the evidence on determinants shaping the optimal selection of vascular access devices, using the COM‐B behavioural model as the theoretical framework.

**Design:**

Systematic review of studies which explore decision‐making at the time of selecting vascular access devices.

**Data Sources:**

The Medline, Web of Science, Scopus and EbscoHost databases were interrogated to extract manuscripts published up to 31 December 2021, in English or Spanish.

**Results:**

Among 16 studies included in the review, 8/16 (50%) focused on physical capability, 8/16 (50%) psychological capability, 15/16 (94%) physical opportunity, 12/16 (75%) social opportunity, 1/16 (6%) reflective motivation and 0/16 (0%) automatic motivation. This distribution represents a large gap in terms of interpersonal and motivational influences and cultural and social environments. Specialist teams (teams created for the insertion or maintenance of vascular access devices) are core for the optimal selection of vascular access devices (75% physical capability, 62% psychological capability, 80% physical opportunity and 100% social opportunity).

**Conclusion:**

Specialist teams predominantly lead all actions undertaken towards the optimal selection of vascular access devices. These actions primarily centre on assessing opportunity and capability, often overlooking motivational influences and social environments.

**Implications for the Profession and/or Patient Care:**

A more implementation‐focused professional approach could decrease inequity among patients and complications associated with vascular access devices.

**Impact:**

- Optimal selection of vascular access devices is the primary strategy in mitigating complications associated with these devices.
- There is a significant disparity between interpersonal and motivational influences and the cultural and social environments. Furthermore, specialized teams play a pivotal role in facilitating the optimal selection of vascular access devices.
- The study can benefit institutions concerned about vascular access devices and their complications.

**Reporting Method:**

This review followed the Preferred Reporting Items for Systematic Reviews and Meta‐Analyses (PRISMA) guidelines.

**Patient or public contribution:**

No Patient or Public Contribution.

**What does this article contribute to the wider global clinical community?:**

- Optimal selection of vascular devices remains a growing yet unresolved issue with costly clinical and patient experience impact.
- Interventions to improve the optimal selection of vascular devices have focused on training, education, algorithms and implementation of specialist vascular teams; alas, these approaches do not seem to have substantially addressed the problem.
- Specialist vascular teams should evolve and pivot towards leading the implementation of quality improvement interventions, optimizing resource use and enhancing their role.

## 1 INTRODUCTION

Catheters are devices used for the administration of drugs, fluids, and blood products. Approximately ¾ of patients would require at least one vascular access device (VAD) during hospital admission (Alexandrou et al., 2018). VADs are selected according to multiple factors such as clinical diagnosis, the status of the patient's vascular system, duration and characteristics of the therapy, availability of devices, patient preferences, and institutional culture and norms among others. Additionally, the knowledge and skill of healthcare professionals to select and insert the most appropriate device are essential (Aizpuru‐Martinez et al., 2021; Enriquez de la Luna‐Rodriguez et al., 2017; Gorski et al., 2021; Grupo de trabajo de la Guía de Práctica Clínica sobre Terapia Intravenosa con Dispositivos no Permanentes en Adultos, 2014; Hallam et al., 2021; Mermel et al., 2001, 2009; Moureau & Carr, 2018; O'Grady et al., 2011; Registered Nurses Association of Ontario, 2005). Furthermore, the selection of VADs occurs at different times during care, with professionals engaging each time with decisions to keep, remove, or re‐insert a device. Perhaps reflecting the complexity of factors and steps embedded in device selection, an agreed definition of optimal selection of VADs is currently lacking.

Selecting an optimal device is not a menial activity, as these devices may cause a range of complications, from mechanical and chemical including phlebitis and extravasations, to infectious, leading to poor care experience and severe adverse events (Zingg et al., 2014). The drivers of these complications are multiple; mechanical incidents may result from an inadequate fit of the device to the size of the vessel, whilst chemical incidents are encouraged by devices unsuitable for the medication administered (Manrique‐Rodriguez et al., 2021), and finally, poor general and cutaneous asepsis contributes to care‐associated infections (Raad et al., 2007). These complications could occur at any point during the insertion, management, maintenance, or removal of VADs.

Strategies to reduce VAD‐related complications start with the optimal selection of VAD, considering the type of device, followed by skilled cannulation techniques under aseptic conditions (Tripathi et al., 2021) on a suitable anatomical site, and responding to the dynamic process of care which juggles clinical demands, medication to be administered, patient characteristics, and clinical response, all which do not remain constant (Blanco‐Mavillard et al., 2022).

## 2 THE REVIEW

Owing to the extensive range of steps and factors aforementioned, the optimal selection of VAD is likely to be driven not only by rational decision‐making (Henderson & Nutt, 1980) but also depend on many other behavioural and human factors (Sax & Clack, 2015). To understand the constellation and relation of influences on the optimal selection of VAD, it would be beneficial to apply a behavioural model. In this regard, the Capability, Opportunity, Motivation, Behaviour (COM‐B) model offers a comprehensive theoretical framework (Michie et al., 2011, 2014; Willmott et al., 2021) which explains any behaviour as the output of the interaction of three components: capability, opportunity and motivation (Table 1).

**TABLE 1:** COM‐B components, definition and examples (Michie et al., 2011).

COM‐B model component and subcomponents		Definition	Example
C	Physical capability	Physical skill, strength or stamina	Having the skill to insert a VAD
	Psychological capability	Knowledge or psychological skills, strength, or stamina to engage in the necessary mental processes	Understanding which is the more appropriate VAD in each situation
O	Physical opportunity	Opportunity afforded by the environment involving time, resources, locations, cues, physical ‘affordance’	Being able to insert a VAD because an ultrasound machine is available in the institution
	Social opportunity	Opportunity afforded by interpersonal influences, social cues and cultural norms that influence the way that we think about things, e.g., the words and concepts that make up our language	Being able to insert the most optimal VAD because your peers understand what you do and support it
M	Reflective motivation	Reflective processes involving plans (self‐conscious intentions) and evaluations (beliefs about what is good and bad)	Intending to select the most optimal VAD
	Automatic motivation	Automatic processes involving emotional reactions, desires (wants and needs), impulses, inhibitions, drive states and reflex responses	Feeling comfortable with oneself at the prospect of being able to insert the most optimal VAD

Therefore, the authors aimed to systematically review the extant literature on the optimal selection of VAD under the COM‐B lens to investigate how decisions are made along the continuum of care to inform interventions to improve clinical performance and mitigate adverse events.

## 3 AIMS

To synthesize the evidence on determinants shaping the optimal selection of VADs, using the COM‐B behavioural model as a theoretical framework, and identifying components vital for decision‐making.

## 4 METHODS

### 4.1 Design

A systematic review of all published studies –quantitative, qualitative, and mixed designs – which explore decision‐making at the time of selecting VADs. The review followed the Preferred Reporting Items for Systematic Reviews and Meta‐Analyses (PRISMA) guidelines (Moher et al., 2009). The study protocol was registered in PROSPERO (CRD42022340905).

### 4.2 Search strategy

The terms used to perform the search were broad to incorporate all studies with relevant information. The keywords and search terms used are available in Appendix 1 **–** Table A1, with the search strategy in Appendix 1 **–** Table A2. The Medline, Web of Science, Scopus and EbscoHost databases (including Psychinfo) were interrogated to extract manuscripts published from 1 January 1900 to 31 December 2021, in English or Spanish. The references of the reviewed studies were searched seeking any other relevant studies.

### 4.3 Study screening, selection and reporting

The authors selected studies in any clinical setting, including outpatients, or where care was provided such as home care, long‐term care facilities and nursing homes and hospices, where healthcare professionals performed any action required for the optimal selection of VAD.

Authors definition of optimal selection of VAD was the choice of an ideal device. This choice is according to patient's characteristics, treatment requirements and clinical stage. Actions which were not performed by health professionals or by patients are excluded. After deduplication, one author screened articles based on titles and abstracts. Following this initial screening, two authors evaluated the full text of the manuscripts for inclusion. The outcomes of the different studies were reported according to the COM‐B categories noted in Table 1.

### 4.4 Search outcome

Actions related to optimal VAD selection were extracted from the selected studies and organized according to the COM‐B model.

### 4.5 Quality appraisal

The authors used the Integrated Quality Criteria for the Review of Multiple Study Designs (ICROMS) tool to assess any biases in the studies (Zingg et al., 2016). This tool allows reviewing of the quality criteria of studies of both quantitative and qualitative methodology. ICROMS tool has two parts: (1) a list of quality criteria specific for each study design, as well as criteria applicable to all study designs using a scoring system; and (2) a decision matrix that specifies the robustness of the study by identifying minimum criteria according to the type of study and the relevance of the study to the research question (Zingg et al., 2016). This tool is composed of 33 items divided into seven categories: (1) Clear aims and justification; (2) Managing bias in sampling or between groups; (3) Managing bias in outcome measurements and blinding; (4) Managing bias in follow‐up; (5) Managing bias in other study aspects; (6) Analytical rigour and (7) Managing bias in report/ethical considerations. ICROMS allows reviewers to score a study depending on the methodology when it successfully meets each criterion: 2 points if a criterion is met; 1 if it is unclear whether or not the criterion is met and 0 if it is not met (Zingg et al., 2016). Studies are then included or excluded if they meet both an overall score and achieve a minimum score threshold in specific categories, depending on the study design evaluated.

However, in the current review, the researchers did not exclude any studies based on the ICROMS score, as the focus was on the actions performed and domains of practice rather than the results obtained in the literature reviewed.

## 5 RESULTS

**FIGURE 1:**
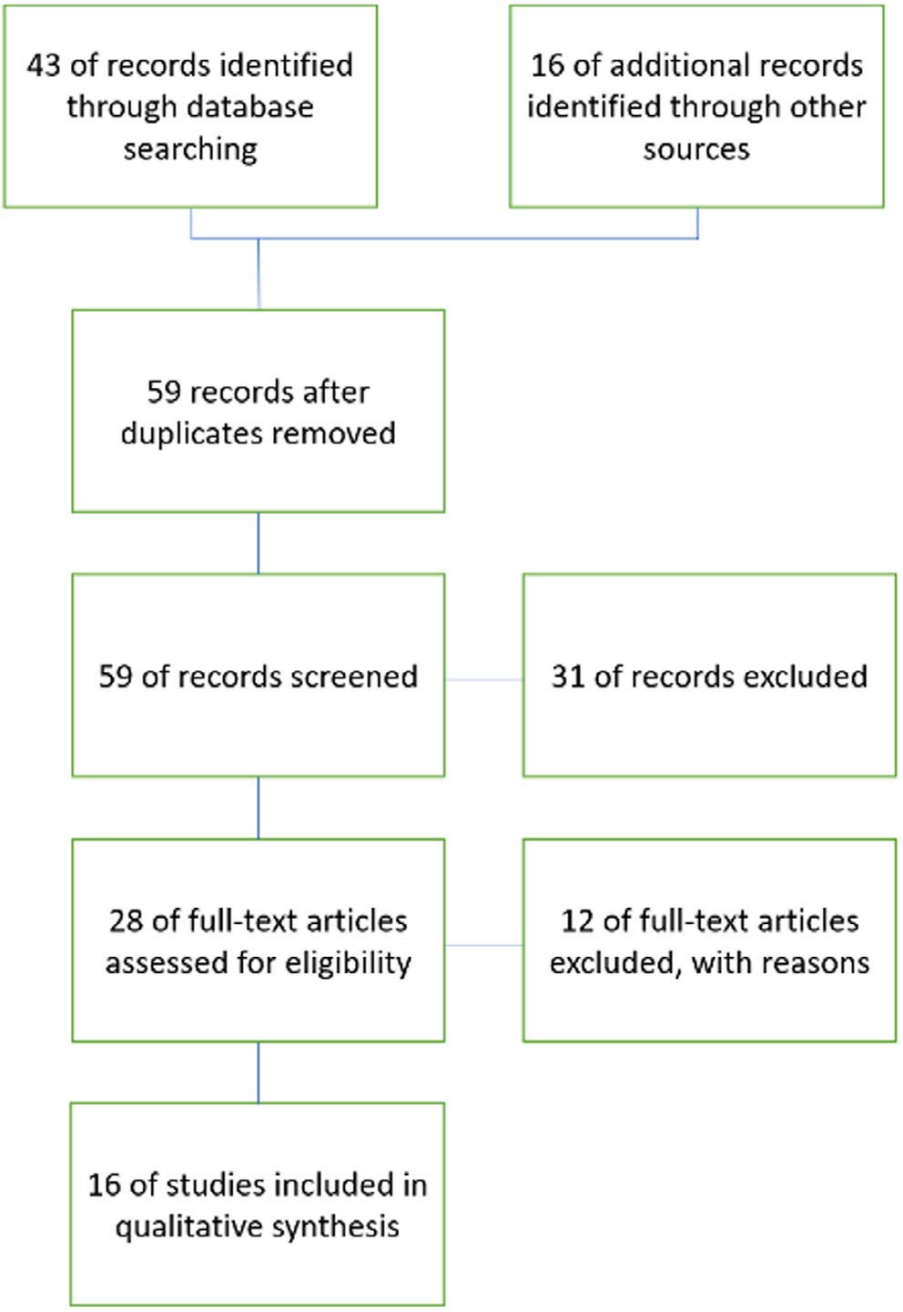
PRISMA diagram.

The initial search retrieved 59 unique articles. The authors excluded 31 articles following a review of the title and abstract. They finally performed a full‐text review of 28 articles, excluding 12 of them (excluded articles with reasons for exclusion in Appendix 2 **–** Table B1). The following results refer to the remaining 16 articles (Figure 1).

Of those 16 studies identified, seven were conducted in the United States, four in Italy, and one each in the Netherlands, United Kingdom, Ireland, Brazil, and Canada. The studies were conducted between 1992 and 2020. Regarding the patient population, 15 studies focused on adults, and one on paediatrics. All but one studies were conducted in hospitals. The characteristics of the studies are presented in Table 2. Table 3 presents the domains of the COM‐B model reflected by each of the studies, where green colour denotes the presence of the item in the article, whilst grey colour indicates that the item is absent. The complete results can be found in Appendix 3.

**TABLE 2:** Characteristics of articles included in review.

Author	Year	Country	Design	Setting	Title
Bell and Spencer (2021)	2020	United States	Observational	Hospital (Emergency Department, 82.3%)	Implementing an emergency department vascular access team: a quality review of training, competency and outcomes
Girotto et al. (2020)	2020	Italy	Cross‐sectional	Paediatric Emergency Department	External validation of the DIVA and DIVA3 clinical predictive rules to identify difficult intravenous access in paediatric patients
Martillo et al. (2020)	2019	United States	Observational	Hospital	A comprehensive vascular access service can reduce catheter‐associated bloodstream infections and promote the appropriate use of vascular access devices
Savage et al. (2019)	2019	United States	Observational	Hospital	Implementation of a Vascular Access Team to reduce central line usage and prevent Central line‐associated bloodstream infections
Civetta et al. (2019)	2018	Italy	Observational	Adult Surgical Patients	Enhanced Adult DIVA score: a new scale to predict difficult preoperative venous cannulation in adult surgical patients
Swaminathan et al. (2018)	2017	United States	Quasi‐experimental, interrupted time series^a^	10 hospitals	Improving PICC use and outcomes in hospitalized patients: an interrupted time series study using MAGIC criteria
Loon et al. (2016)	2016	Netherlands	Observational, Cross‐sectional Cohort	Department of Anesthesiology, Adult patients	Development of the A‐DIVA scale: a clinical predictive scale to identify difficult intravenous access in adult patients based on clinical observations
Pagnutti et al. (2016)	2015	Italy	Pilot‐validation^b^	Adults undergoing single chemotherapy cycles	Difficult intravenous access tool in patients receiving peripheral chemotherapy: a pilot‐validation study
Bellesi et al. (2013)	2013	Italy	Observational	Patients with autologous peripheral blood stem cell transplantation procedures	PICCs in the management of onco‐haematological patients submitted to autologous stem cell transplantation
Barr et al. (2012)	2012	United Kingdom	Retrospective Cohort	Outpatient parenteral antimicrobial therapy	Self‐administration of outpatient parenteral antibiotic therapy and risk of catheter‐related adverse events: a retrospective cohort study
Carr et al. (2010)	2010	Ireland	Observational	Hospital	A pilot intravenous cannulation team: an Irish perspective
da Silva et al. (2010)	2010	Brazil	Observational	Hospital admission units covering internal medicine, surgery, haematology, and oncology	Benefits of establishing an intravenous team and the standardization of peripheral intravenous catheters
Bosma and Jewesson (2002)	2002	Canada	Observational	Hospital	An infusion program resource nurse consult service: our experience in major Canadian teaching hospital
Palefski and Stoddard (2001)	2001	United States	Observational Prospective evaluation	Two hospitals and one home infusion agency	The infusion nurse and patient complication rates of peripheral‐short catheters. A prospective evaluation
Meier et al. (1998)	1998	United States	Observational Before‐after	Hospital	Impact of a dedicated intravenous therapy team on nosocomial bloodstream infection rates
Scalley et al. (1992)	1992	United States	Observational	Hospital	The impact of an intravenous therapy team on the occurrence of intravenous‐related phlebitis. A 30‐month study

^a^ Considered as controlled before‐after study in the analysis of bias.

^b^ Considered as observational study in the analysis of bias.

**TABLE 3:** Data extraction according to COM‐B model.

Studies	COM‐B										
	Capability				Opportunity				Motivation		
	Physical capability		Psychological capability		Physical opportunity		Social opportunity		Reflective motivation		Automatic motivation
Team composition	Spec	Gen	Spec	Gen	Spec	Gen	Spec	Gen	Spec	Gen	
Bell and Spencer (2021)	U/S Training		Education		Assuming insertion	C‐Diva Scale	Social support				
Girotto et al. (2020)		U/S Training				DIVA & DIVA3 scale					
Martillo et al. (2020)					Assuming insertion Algorithms		Social support				
Savage et al. (2019)	U/S Training		Education	Education	Assuming insertion		Social support		Explains the advantages of having standardized practice		
Civetta et al. (2019)					EA‐DIVA scale Assuming insertion		Social support				
Swaminathan et al. (2018)	Training		Education	Education	Assuming insertion MAGIC based tool Recommendations in electronical medical record		Social support				
Loon et al. (2016)						Puncture difficulty scale					
Pagnutti et al. (2016)						Puncture difficulty scale U/S					
Bellesi et al. (2013)					Assuming insertion		Social support				
Barr et al. (2012)					Assuming insertion Algorithms		Social support				
Carr et al. (2010)	Training				Assuming insertion		Social support				
da Silva et al. (2010)				Education	Assuming insertion		Social support				
Bosma and Jewesson (2002)		Training		Education	Assuming insertion		Social support				
Palefski and Stoddard (2001)	Selection made by skills		Selection made by experience								
Meier et al. (1998)				Education	Assuming insertion		Social support				
Scalley et al. (1992)	Training	Training	Education	Education	Assuming insertion		Social support				
**Subtotal items**	**6/8**	**4/8**	**5/8**	**7/8**	**12/15**	**4/15**	**12/12**		**1/1**		
**Total items present**	**8/16**		**8/16**		**15/16**		**12/16**		**1/16**		**0/16**

Due to the high frequency of occurrence of specialist teams (teams created for the insertion and maintenance of vascular access devices, whether they are composed of one or several professionals and from any discipline) in relation to their representation in healthcare settings (Carr et al., 2018), (physical capability 75%, psychological capability 62% and physical opportunity 80%) in Table 3 are represented separately.

### 5.1 Physical capability

This item broadly refers to ‘physical skill, strength or stamina’ (Michie et al., 2014). Of the eight articles identified which included an element related to physical capability (Bell & Spencer, 2021; Bosma & Jewesson, 2002; Carr et al., 2010; Girotto et al., 2020; Palefski & Stoddard, 2001; Savage et al., 2019; Scalley et al., 1992; Swaminathan et al., 2018), three reported on the training in handheld ultrasound scanners to aid VAD insertion and accompaniment in successful punctures, training by a nurse employed by the company that sells peripherally inserted central catheters (PICCs) and midlines on ultrasound‐guided insertions (Bell & Spencer, 2021; Girotto et al., 2020; Martillo et al., 2020; Savage et al., 2019), with four of them documenting training in PICC insertion, midlines, alternative peripheral VAD devices, cannulation, training and supervision about clinical performance on insertions (Bosma & Jewesson, 2002; Carr et al., 2010; Scalley et al., 1992; Swaminathan et al., 2018).

### 5.2 Psychological capability

This item refers to ‘knowledge or psychological skills, strength or stamina to engage in necessary mental processes’ (Michie et al., 2014). The eight articles related to psychological capability (Bell & Spencer, 2021; Bosma & Jewesson, 2002; da Silva et al., 2010; Meier et al., 1998; Palefski & Stoddard, 2001; Savage et al., 2019; Scalley et al., 1992; Swaminathan et al., 2018) showcase different education programs, for example, related to anatomy, vascular access and ultrasound, evidence‐based practice review, guidelines (Chopra et al., 2015), vascular access device selection, care, maintenance and removal, appropriate vascular access device selection, standardize insertion, clinical, educational and research support.

### 5.3 Physical opportunity

This item focuses on ‘opportunity afforded by the environment involving time, resources, locations, cues, physical ‘affordance” (Michie et al., 2014). In the 15 studies identified in the review (Barr et al., 2012; Bell & Spencer, 2021; Bellesi et al., 2013; Bosma & Jewesson, 2002; Carr et al., 2010; Civetta et al., 2019; da Silva et al., 2010; Girotto et al., 2020; Loon et al., 2016; Martillo et al., 2020; Meier et al., 1998; Pagnutti et al., 2016; Savage et al., 2019; Scalley et al., 1992; Swaminathan et al., 2018), 12 explored the physical resources (time, materials, devices) available to teams of specialists in vascular access to perform an optimal selection or insertion of the VAD (Barr et al., 2012; Bell & Spencer, 2021; Bellesi et al., 2013; Bosma & Jewesson, 2002; Carr et al., 2010; Civetta et al., 2019; da Silva et al., 2010; Martillo et al., 2020; Meier et al., 1998; Savage et al., 2019; Scalley et al., 1992; Swaminathan et al., 2018), whilst three studies reported on algorithms to aid the optimal selection of VAD (Barr et al., 2012; Martillo et al., 2020; Swaminathan et al., 2018) and finally five of them documenting the effect of different scales to assess difficult venipuncture and help with the optimal selection of the VAD or insertion technique (Bell & Spencer, 2021; Civetta et al., 2019; Girotto et al., 2020; Loon et al., 2016; Pagnutti et al., 2016).

### 5.4 Social opportunity

This item discusses the ‘opportunity afforded by interpersonal influences, social cues, and cultural norms that influence the way people think about things, such as the words and concepts that make up their language’ (Michie et al., 2014). The 12 studies (Barr et al., 2012; Bell & Spencer, 2021; Bellesi et al., 2013; Bosma & Jewesson, 2002; Carr et al., 2010; Civetta et al., 2019; da Silva et al., 2010; Martillo et al., 2020; Meier et al., 1998; Savage et al., 2019; Scalley et al., 1992; Swaminathan et al., 2018) where this indicator was identified centre on teams of specialists in vascular access – although with different nomenclatures or naming – and how they support and influence different behaviours related to insertion and maintenance of VAD.

### 5.5 Reflective motivation – Automatic motivation

These items refers to ‘reflective processes involving plans (self‐conscious intentions) and evaluations (beliefs about what is good and bad)’, and ‘automatic processes involving emotional reactions, desires (wants and needs), impulses, inhibitions, drive states and reflex responses’ (Michie et al., 2014). Only one study (Savage et al., 2019) referred to reflective motivation, where a specialist team explained to physicians and nurses why standardized practices were needed to reduce unnecessary central catheter use and minimize the risk of infection.

## 6 QUALITY ASSESSMENT

The ICROMS dimensions and scores are presented in Table 4. In this review, the quality of the studies evaluated was variable. It is worth noting that whilst the worst‐rated study is the most recent (Bell & Spencer, 2021), the only one which complies with all the items in the scale is from 2012 (Barr et al., 2012).

**TABLE 4:** ICROMS quality assessment scores.

Studies	Icroms dimension						
	Clear aims and justification	Managing bias in sampling or between groups	Managin bias in outcome measurements and blinding	Managing bias in follow‐up	Managing bias in other study aspects	Analytical rigour	Managing bias in reporting/ethical considerations
Bell and Spencer (2021)	2/6	0/2	0/4	0/2	0/4	1/2	2/10
Girotto et al. (2020)	6/6	2/2	2/4	0/2	2/4	2/2	9/10
Martillo et al. (2020)	5/6	2/2	4/4	0/2	0/4	2/2	6/10
Savage et al. (2019)	5/6	2/2	4/4	0/2	3/4	1/2	4/10
Civetta et al. (2019)	2/2	2/2	4/4	1/2	4/4	2/2	8/10
Swaminathan et al. (2018)	2/2	1/2	4/8	0/2	2/2	2/2	8/10
Loon et al. (2016)	2/2	2/2	4/6	2/2	2/2	1/2	5/10
Pagnutti et al. (2016)	1/6	0/2	2/4	0/2	0/4	2/2	6/10
Bellesi et al. (2013)	2/6	0/2	4/4	0/2	1/4	2/2	4/10
Barr et al. (2012)	2/2	2/2	6/6	2/2	2/2	2/2	9/10
Carr et al. (2010)	2/6	0/2	3/4	1/2	0/4	2/2	3/10
da Silva et al. (2010)	0/6	0/2	4/4	0/2	1/4	0/2	0/10
Bosma and Jewesson (2002)	1/6	0/2	0/4	1/2	2/4	2/2	3/10
Palefski and Stoddard (2001)	2/6	0/2	2/4	1/2	0/4	2/2	5/10
Meier et al. (1998)	2/6	2/2	4/4	0/2	2/4	2/2	5/10
Scalley et al. (1992)	2/6	2/2	4/4	0/2	2/4	1/2	3/10

## 7 DISCUSSION

Over the next few years, the suboptimal selection of VAD is likely to increase. Nowadays, people over 65 years old have a larger average length of hospital stays and a higher frequency of hospital admissions (Eurostat Statistics Explained, 2023). If this trend persists, peripheral intravenous catheters, which are the indicated devices for not irritating treatments that last for less than 7 days, might not be the most suitable devices for this growing segment of the population. Moreover, as drug‐resistant bacteria spread, longer and potentially more irritating treatments might be necessary (CDC, 2019; Jarzebski et al., 2021). To the best of the authors' knowledge, this is the first systematic synthesis of the evidence underpinning clinical decisions about VAD selection and insertion from the perspective of behavioural determinants advocated by the COM‐B model.

Capability, opportunity and motivation can be influenced by different strategies (Michie et al., 2011). In half of the studies included in the review, capability was influenced by knowledge and skill. Physical capability was influenced by skill as in training to use ultrasound or other imaging devices, with psychological capability influenced by knowledge as education for optimal selection of VAD, anatomy, ultrasound, and guidelines. These findings are consistent with recommendations in different clinical guidelines, international consensus like Reference study (Blanco‐Mavillard et al., 2023), and GaVeCeLT (Gli Accessi Venosi Centrali a Lungo Termine, which is a multidisciplinary and multiprofessional team dedicated to central vascular access) recommendations to train professionals on insertion and optimal selection of VAD (Gorski et al., 2021).

As for opportunity, more than 80% of the studies found it was influenced by time, resources, location and physical barriers, through specialist teams, algorithms and scales about difficulty‐in‐punction. These algorithms are like those proposed in the Michigan Appropriateness Guide for Intravenous Catheters (MAGIC) guidelines (Chopra et al., 2015; Ullman et al., 2020), but none of them include shared decision‐making with patients. As for motivation, in most of the studies reviewed, nothing is done to influence it. Reflexive and automatic motivation seem difficult to explore via quantitative methodologies, and the authors did not find any qualitative papers. It is not surprising that the evidence identified was chiefly focused on high‐resource countries (Global Minority), as the teams of specialist nurses whom most actions centre upon require both material and personal resources. In this regard, most studies were carried out in hospital settings and in adults, with little or no sample from home settings and long‐stay units.

The different aspects related to the selection of VAD have been explored from the point of view of academics and healthcare professionals. However, they also ought to be explored from the standpoint of decision‐makers, patients, specialist teams and generalist teams, in short, a wider range of stakeholders. Any behavioural model and theory would serve this purpose, but the COM‐B model allows the identification, in a simple way, of gaps which may then be easily adaptable to the Theoretical Domains Framework (TDF), and converted into interventions (behaviour change techniques) ultimately transferable to policies (Michie et al., 2014).

The accurate assessment of non‐optimal VAD selection as an area for improvement remains unresolved due to a lack of an operational and agreed‐upon definition (Carr et al., 2018). In the last 20 years different solutions have been proposed to address optimal VAD selection, including algorithms (Barr et al., 2012; Civetta et al., 2019; Martillo et al., 2020; Swaminathan et al., 2018), recommendations (Chopra et al., 2015; Ullman et al., 2020), or integration of specialist teams and training in ultrasound techniques for VAD insertion (Barr et al., 2012; Bell & Spencer, 2021; Bellesi et al., 2013; Bosma & Jewesson, 2002; Carr et al., 2010; Civetta et al., 2019; da Silva et al., 2010; Martillo et al., 2020; Meier et al., 1998; Savage et al., 2019; Scalley et al., 1992; Swaminathan et al., 2018). However, it is unclear if these strategies based on training and education have substantially altered the landscape of optimal VAD selection in clinical practice. Linking back to the COM‐B model, physical and social opportunities are usually addressed by specialist teams (Carr et al., 2018), algorithms, and scales. However, interpersonal and motivational influences as well as cultural and social environments are hardly addressed due to the difficulty in measuring them. Nowadays, specialist teams seem to oversee the optimal selection, insertion, management and maintenance of VADs, which may lead to inequities in the care received by some patients without access to these specialist teams. Another potential unintended consequence of this prominence of specialist teams might be the loss of an integrated approach to VAD care by the generalist team, detached from VAD selection, insertion or maintenance, and therefore prone to deliver fragmented and ‘taskified’ care (Blanco‐Mavillard et al., 2022; Castro‐Sanchez et al., 2014).

Many specialist teams, currently nurse‐led, were developed to relieve the work of medical specialists in the insertion of VADs like central venous catheters. This task‐shifting approach aimed to optimize use of resources whilst maintaining quality standards (van Schalkwyk et al., 2020). The consolidation of this model of care worldwide (Cortes Rey et al., 2021) affords opportunities to consider further and future expansions of roles and competencies among this highly specialized cadre of nurses leading vascular access care. It may soon for example make little sense for these teams to focus only, or chiefly, on VAD insertion. Instead, the optimal selection of VAD and its insertion may be a task performed in principle by the generalist team. In more complex or difficult cases, the specialist VAD teams would then step in to aid in the selection and insertion of devices.

Even better use of resources would be possible if specialist teams were leaders in the implementation of quality improvement interventions in clinical settings, which is a complex and multifaceted phenomenon that requires adequate competencies (Harvey et al., 2002; Nilsen, 2015). Whilst the professionals in specialist teams currently have the knowledge and skills to train and educate peers and other healthcare workers, as well as carers and patients, implementation competencies and proficiency among these teams are yet to be determined. A model to address the optimal selection of VAD in which specialist teams were to adopt roles related to implementation would reach more patients, increasing equity and decreasing the costs derived from the non‐optimal selection of VAD.

## 8 STRENGTHS AND LIMITATIONS

The study has strengths, including the use of a theoretical framework that provides robustness analysing the results and facilitates the comparison of findings with future studies. Among the limitations of this review, terms in the COM‐B model are unclear in relation to the context. Furthermore, the different study designs considered make it challenging to integrate findings, compounded by the variable methodological quality of the articles finally included in the review. Finally, the broad search strategy reflects the current lack of agreement on the definition of optimal VAD selection.

## 9 CONCLUSION

In the last 20 years, there seems to have been no change in the approach towards the problem of optimal selection of VAD, as more than a half of studies have actions focused on capability, and almost all studies have some sort of action focused on opportunity yet only one study has some kind of action about motivation. Interpersonal and motivational influences and cultural and social environments are alas hardly addressed.

Specialist teams remain at the forefront of vascular access care including optimal selection of VAD. Now it may be a good time to consider whether these teams should pivot towards leading implementation interventions and efforts targeting interpersonal and behavioural contexts as well as sociocultural environments influencing vascular access device management and care.

## AUTHOR CONTRIBUTIONS

IF‐F is the principal investigator of the study. All authors contributed to the original idea, design of the study, and are responsible for the conduct of the study. IF‐F prepared the first draft of the manuscript. All authors provided critical commentary on drafts and approved the final protocol manuscript.

## FUNDING INFORMATION

This research received no specific grant from any funding agency in the public, commercial or not‐for‐profit sectors.

## CONFLICT OF INTEREST STATEMENT

The authors declare that they have no competing interests.

## PEER REVIEW

The peer review history for this article is available at https://www.webofscience.com/api/gateway/wos/peer‐review/10.1111/jan.16202.

## CONSENT FOR PUBLICATION

This manuscript does not contain data from any individual person.

## PROTOCOL REGISTRATION

Prospero (International prospective register of systematic reviews). Registration: CRD42022340905 https://www.crd.york.ac.uk/prospero/display_record.php?RecordID=340905.

## Data Availability

Data sharing is not applicable to this article as no new data were created or analysed in this study.

## APPENDIX 1 SEARCH STRATEGY

**TABLE A1:** Terms of search strategy.

	Mesh	Decs	Free term
1	Vascular access devices	Vascular access devices	
2	Catheterization, peripheral		Peripheral venous catheter
3			Short peripheral venous catheter
4			Long peripheral venous catheter
5	Central venous catheter		Central venous catheter
6			Tunnelled central venous catheter
7			Non‐tunnelled central venous catheter
8	Catheterization, peripheral		Peripheral inserted central catheter – PICC
9			Short duration PICC
10			Long duration PICC
11			Tunnelled PICC
12			Mid‐line catheter
13			Micro mid‐line catheter
14			Mini mid‐line catheter
15			Mid‐clavicular mid‐line
16			Subcutaneous reservoir catheter
17	Vascular access device		Port‐a‐cath
18			PICC‐Port
19	Decision making Clinical decision making Decision making, organizational Decision making, computer‐assisted Decision making, shared	Decision making	
20			Adequacy
21			Ph Adequacy
22			Osmolarity adequacy
23			Treatment irritability adequacy
24			Treatment vesicance adequacy
25			Treatment toxicity adequacy
26			Scales
27	Logistic Models		
28			Common practice
29			Number of punctures
30			VAD duration
31			Time to start treatment
32			Delayed start of treatment
33			Missed dose of treatment
34			Number of VAD during admission
35	Vascular access device – adverse effects		Complications derived from the use of VAD

**TABLE A2:** Search strategy.

SEARCH STRATEGY				
(1–18 OR)	AND	(19–28 OR)		
(1–18 OR)	AND	(29–35 OR)		
(1–18 OR)	AND	(19–28 OR)	AND	(29–35 OR)
Language filter (Spanish–English)				
Use of terms: 1 – Mesh 2 – Decs (if Mesh not available or Mesh already used) 3 – Free term (if Decs or Mesh not available or already used)				

## APPENDIX 2 EXCLUDED ARTICLES

**TABLE B1:** Excluded articles and reasons.

Nicholas et al. (2021)	It compares two navigation models. It does not discuss the suitability of these devices or improve the material available; it simply compares two products with the same function.
Ullman et al. (2020)	It does not meet inclusion criteria, as it is not a professional using any method of vascular device selection or any patient receiving the device.
Nibbelink and Brewer (2018)	Identifies factors and processes related to patient care decision making in medical‐surgical settings.
Kang et al. (2017)	Multicentre follow‐up of catheters for complications.
Burbridge et al. (2016)	A self‐designed satisfaction scale is carried out on the armports.
Stolz et al. (2016)	They evaluate the learning curve of ultrasound‐guided puncture. They establish that at least 4 insertions must be performed for a first puncture success rate of 70%.
Chopra et al. (2016)	A prospective study at 10 hospitals with patients who received PICC, not declaring the use of any method of vascular device selection.
Chopra et al. (2015)	It does not meet inclusion criteria, as it is not a professional using any method of vascular device selection or any patient receiving the device.
Wu et al. (2014)	It talks about port insertion locations (Port‐A‐Cath) but nothing that leads them to select that device.
Botella‐Carretero et al. (2013)	It only compares two devices, although it could fall into the O of opportunity does not seem a specific material for the correct selection of the vascular access device.
Soifer et al. (1998)	Compares catheters inserted by generalists and VAST (vascular access specialist team). Patients are randomized based on medical history number. They do not function as an aid to proper vascular access device selection.
Tomford et al. (1984)	They do nothing for the correct selection of the vascular access device. They simply assign units in which they insert VAST (when available 40 h/week) and units in which they insert generalists.

*Note*: Botella‐Carretero et al. (2013); Burbridge et al. (2016); Chopra et al. (2015, 2016); Kang et al. (2017); Nibbelink and Brewer (2018); Nicholas et al. (2021); Soifer et al. (1998); Stolz et al. (2016); Tomford et al. (1984); Ullman et al. (2020); Wu et al. (2014).

## APPENDIX 3 DATA EXTRACTION

### Physical capability

1. Training of professionals of the emergency department vascular access team, in ultrasound‐guided puncture and accompaniment in 3 successful punctures (Bell & Spencer, 2021).
2. All nursing staff in the centre receives ultrasound insertion training as a result of another project being carried out at the centre (Girotto et al., 2020).
3. A nurse employed by the company that sells PICCs and midlines conducts training for specialist team members on PICC insertion and ultrasound‐guided insertions. The team then teaches this new procedure to coordinators and educators and validates their competencies. Annual competency evaluations are conducted in central lines and ports maintenance (Savage et al., 2019).
4. Training for vascular access nurses on alternative peripheral venous access devices as recommended by MAGIC (Swaminathan et al., 2018).
5. All intravenous cannulation team members were trained in venipuncture and cannulation. Subsequently, the team leader supervises the members in the performance of 100 insertions (Carr et al., 2010).
6. Infusion program nurses provide training to the rest of nurses in insertion, basic and advanced infusion, as well as just in time training in infusion, insertion and maintenance (Bosma & Jewesson, 2002).
7. Nurses are classified as infusion or generalist based on a manager's decision based on skill and experience criteria. No direct training is provided (Palefski & Stoddard, 2001).
8. Formal orientation checklist and skills list for IVT members. Training for floor nurses. Nurses are given a skills checklist after centre training. When new nurses are hired for the centre, specialist team members review the procedures (Scalley et al., 1992).

### Psychological capability

1. Education program covering anatomy, vascular access and ultrasound, evidence‐based practice review and use of the centre's ultrasound scanners (Bell & Spencer, 2021).
2. A nurse working for the company that supplies PICCs and midlines will provide education to the specialist team members on PICC insertion skills. The specialist team educate physicians and nurses on MAGIC guidelines to reduce unnecessary central catheter insertion, educate new staff on vascular access device selection, care, maintenance and removal (Savage et al., 2019).
3. Provider education regarding the importance of appropriate vascular access device selection (Swaminathan et al., 2018).
4. Education of the hospital infection control service have been made along professionals to standardize care and re‐insertion of peripheral vascular devices (da Silva et al., 2010).
5. The multidisciplinary specialist team gives clinical, educational and research support (Bosma & Jewesson, 2002).
6. Nurses are classified as infusion or generalist based on a manager's decision based on skill and experience criteria. No direct education is provided (Palefski & Stoddard, 2001).
7. The specialist team will be responsible of provide staff education on IV therapy (Meier et al., 1998).
8. Professionals who become part of the specialist team undergo education and orientation. Subsequently, a specialist team conducts education for generalist nurses (Scalley et al., 1992).

### Physical opportunity

1. The C‐DIVA (puncture difficulty scale) was implemented for the use of ED department and specialist team with staff of 5 nurses 24 hours a day in the institution perform insertions (Bell & Spencer, 2021).
2. They want to make an internal protocol for predicting difficulty and refer those patients to the use of US techniques, scales that pass the nurses who have been trained in the use of US to perform a validation of the scales (DIVA, DIVA3) (Girotto et al., 2020).
3. Apart from focusing on performing insertions correctly they also work to insert the most correct device using evidence and algorithms to ensure that patients are given devices that are appropriate to their needs and avoid inserting central access devices that are not necessary. They present several algorithms. One for patients with kidney disease in which the first step of the algorithm is if they need a dialysis access, in a second term they use the staging of the disease, survival, unit in which they are located, functionality of the access if they already have it and prognosis. A second algorithm for oncology patients that considers the pathology, the difficulty of access, the planning of administering vesicant medication, whether extravasation has occurred and the expected duration of treatment all seem to be algorithms focused on being used by the team of specialists. Specialist team is divided into 3 teams depending on whether they want to insert PVAS, CVAS, VAST (Martillo et al., 2020).
4. Specialist team with a staff of 3 nurses. It oversees inserting peripheral catheters, midlines, PICCs and monitoring central catheters and midlines. The team together with the infection control department check central devices daily and force early removal (Savage et al., 2019).
5. Throughout the study, they use a puncture difficulty scale (EA‐DIVA) that they refine until they obtain a scale that with a score higher than 8 points refers you to special techniques or professional specialist, the surgical unit staff (resident, anesthesiologic staff, nurse) to perform the insertion and collect data and with those data create the scale, that scale finally with 8/12 points or more refers you to specialist or special techniques (Civetta et al., 2019).
6. A MAGIC based tool to evaluate appropriateness of PICC placement prior to insertion and changes to the electronic medical record that incorporated MAGIC recommendations (Swaminathan et al., 2018).
7. Puncture difficulty scale with 3 risk groups: low, medium and high (Loon et al., 2016).
8. Specialist team develop a puncture difficulty scale that they propose as useful instrument to know the puncture difficulty of patients receiving chemotherapy by peripheral accesses, ultrasound equipment is available for staff if they have received training in its use (Pagnutti et al., 2016).
9. All PICCs are inserted by a specifically trained PICC TEAM. Maintenance is provided by specially trained nurses (Bellesi et al., 2013).
10. The specialist team who will perform the insertion. Algorithm of choice based on treatment duration, irritancy, use of ultrasound and whether peripheral access is good. (Barr et al., 2012).
11. Specialist team without 24/7 coverage is dedicated to peripherical catheter insertion (Carr et al., 2010).
12. IV insertion team that operates in 6 admission units in the institution (da Silva et al., 2010).
13. The infusion nurses provide consultation 7 days a week. In DIVA situation or in case of device complications the infusion nurse will be able to do something or postpone her decision for PICC insertion (Bosma & Jewesson, 2002).
14. The specialist team performs the insertion and maintenance of the devices (Meier et al., 1998).
15. Specialist team has 10 nurses and insert and monitor all catheters daily (Scalley et al., 1992).

### Social opportunity

1. The emergency department vascular access specialist team performs the function of social support (Bell & Spencer, 2021).
2. Vascular access service comprised of three teams: central venous access service, vascular access tunnelled catheter service and team of specialized vascular access nurses, performs the function of social support (Martillo et al., 2020).
3. Vascular access team performs the function of social support (Savage et al., 2019).
4. The specialist that is referred by the scale for evaluation of the case performs the function of social support (Civetta et al., 2019).
5. The vascular access team performs the function of social support (Swaminathan et al., 2018).
6. The PICC team performs the function of social support (Bellesi et al., 2013).
7. Specialist nurse practitioners perform the function of social support (Barr et al., 2012).
8. Intravenous cannulation team performs the function of social support (Carr et al., 2010).
9. A dedicated intravenous team performs the function of social support (da Silva et al., 2010).
10. Infusion program resource nurse consult service performs the function of social support (Bosma & Jewesson, 2002).
11. Members of a specialized intravenous team perform the function of social support (Meier et al., 1998).
12. Intravenous team performs the function of social support (Scalley et al., 1992).

### Reflective motivation

1. Specialist team explains to physicians and nurses why standardized practices are needed to reduce unnecessary central catheters use and minimize the risk of infection (Savage et al., 2019).

### Automatic motivation

No reference to this item was found in the articles.
